# Dietary Factors Associated with Dental Erosion: A Meta-Analysis

**DOI:** 10.1371/journal.pone.0042626

**Published:** 2012-08-31

**Authors:** Haifeng Li, Yan Zou, Gangqiang Ding

**Affiliations:** 1 Children’s Hospital, Zhejiang University School of Medicine, Hangzhou, China; 2 Zhejiang Provincial Center for Disease Control and Prevention, Hangzhou, China; University of Toronto, Canada

## Abstract

**Background:**

Some diet factors are risk factors for dental erosion.

**Methods:**

We performed computer searches of PubMed, Cochrane Library, EBSCO, CALIS, et al., to search for studies investigating risk factors for dental erosion. For risk factors investigated in a comparative way, we computed pooled odds ratios (ORs) using the Mantel and Haenszel method.

**Results:**

A total of 9 studies met the inclusion criteria, and 6 risk factors were considered, including soft drinks, sports drinks, juice, vitamin C, milk, and yoghourt. The following associations were found for soft drinks (OR = 2.41, 95%CI = 2.03–2.85) and vitamin C (OR = 1.16, 95%CI = 1.10–1.22). While juice (OR = 0.90, 95%CI = 0.25–3.24), sports drinks (OR = 1.58, 95%CI = 0.88–2.85), milk (OR = 0.67, 95%CI = 0.11–4.01), and yoghourt products (OR = 1.05, 95%CI = 0.28–3.96) were not associated with dental erosion.

**Conclusions:**

This meta-analysis provides comprehensive evidence-based assessment of diet-related factors for dental erosion. Preventive strategies should be taken to reduce dental erosion.

## Introduction

Dental erosion is clinically defined as the progressive and irreversible loss of dental hard tissue caused by a chemical process of acid dissolution that does not involve bacteria [1]. According to recent studies, there is some evidence that the presence of dental erosion is steadily increasing [2]. Dental erosion can have extrinsic or intrinsic causes. Extrinsic factors include demineralizing acidic foods-such as citrus fruits and acidic beverages and some medicines-such as effervescent vitamin C preparations, chewable vitamin C tablets [3], [4]. Intrinsic causes of erosion include recurrent vomiting as a result of psychological disorders, e.g., in anorexia and bulimia or regurgitation of gastric contents because of some abnormality in the gastrointestinal tract [5]. One important additional factor in dental erosion is low salivary flow, which, naturally, results in inadequate rinsing and buffering of demineralizing acids on tooth surfaces [6].

There is growing evidence of a considerable increase in consumption of potentially erosive drinks. There have been significant associations shown between soft drink consumption and dental erosion [7]. Many previous reports have found dental erosion to be significantly associated with the diet factors although some have not [8]–[24]. However, the contributions of the associated diet factors to dental erosion are still unclear. Therefore, the aim of this meta-analysis study was to review dietary factors associated with dental erosion, with emphasis on estimation of the quantitative contributions.

## Methods

### Search Strategy and Selection Criteria

As the basis for our analysis, we performed a computer searches of PubMed, Cochrane Library, EBSCO, CALIS, Wanfang Database, China National Knowledge Infrastructure (CNKI) and Weipu Database for the literature form 1992 to December 2011. The search themes were combined as “(carbonated or soft drink or soft drinks) and (water or milk or juice or fruits or diet) and (tooth or teeth or dental) not vitro”. In addition, we performed a Google Scholar search.

Two investigators (YZ, HL) independently reviewed titles and abstracts, and selected articles addressing diet and dental erosion. Disagreements were resolved by discussion and consensus. On a second sift; we selected original studies on diet and dental erosion with the following inclusion criteria :(1)Addressing diet and dental erosion, (2)The study should provide sample size, (3)The study should provide associated factors with odds ratio with interval. Studies were exclude if one of the following existed: (1) Not related to diet and dental erosion, (2) Animal studies, (3) odds ratios or frequency were not reported, and (4) reviews and abstracts.

The computer search produced 417 citations. Records after duplicates removed resulted in 417 citations. Review of titles and abstracts resulted in the selection of 12 papers, among which 9 met the inclusion criteria.

**Figure 1:**
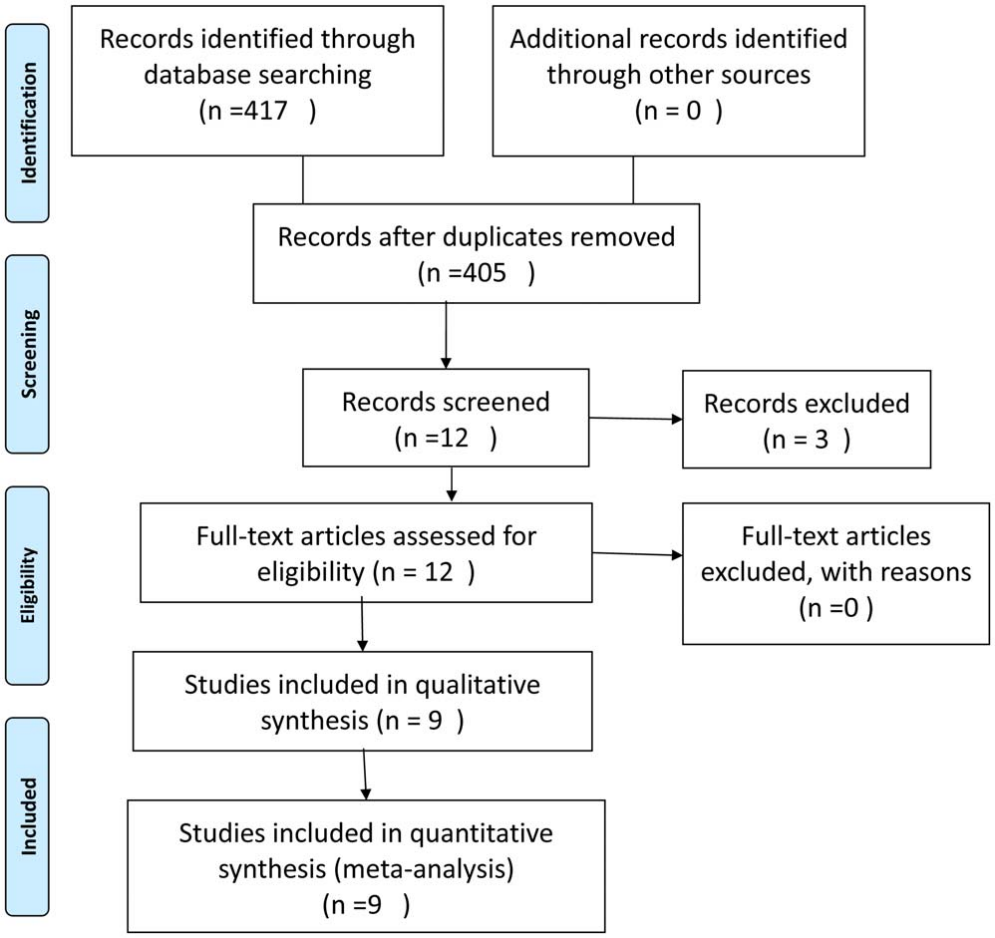
Flow chat of selection of studies for inclusion in meta-analysis.

### Data Extraction

For each study included, the full text was retrieved and the following data were extracted: location, year of publication, size and mean age of the sample. For each associated factors, we extracted the Odds Ratios(ORs) together with confidence intervals (CIs).

Two investigators performed the extraction of the data in duplicate to avoid errors. Multivariate estimates were always selected when available; otherwise the unadjusted results were recorded. We pooled studies that present ORs.

Given the high number of potential associated factors, we restricted our analysis to those for which the risk was assessed by at least 4 studies. Each associated factor was analyzed separately.

**Table 1:** Summary of studies for dietary factors associated with dental erosion.

Study	Author, Reference	Year	Location	Sample Size Number	Female(%)	Mean or Median Age of Study Population
1	Nahás Pires Corrêa MS et al [16]	2011	Brazil	232	50	9.5
2	Aidi HE et al [17]	2011	Netherlands	572	49	11.9
3	Ratnayake N et al [18]	2009	Sri Lanka	1200	53	17
4	Chen YG et al [19]	2009	China	2005	48	5,12
5	Waterhouse PJ et al [20]	2008	UK	458	NR	13.8
6	Dugmore CR et al [21]	2004	UK	1753	49	12
7	Milosevic A et al [22]	2004	UK	2385	52	14
8	Tarsitsa Gatou et al [23]	2011	GREECE	198	50	6.37
9	Milosevic A, et al [24]	1997	UK	74	NR	15

NR indicates not reported.

### Statistical Analysis

We used SAS (r) Software 9.2 to analyze data. We assessed statistical heterogeneity among studies using the χ^2^ test and I^2^ statistics was calculated to quantify the proportion of the total variation due to heterogeneity. We estimated ORs and 95% CIs using Mantel and Haenszel method [25] using generic inverse variance method [26] when the P value >0.05 for the Q test which indicated a lack of heterogeneity among the studies. Forest plots were created, which show the effect estimate, level of variability around that estimate for each study and the weight given to each study in the meta-analysis along with the overall pooled result. Statistical significance was set at P<0.05 and 95% confidence intervals are quoted throughout. The two authors inputted the data in the statistic software SAS (r) Software 9.2 to perform the statistical analysis in dependently and got the same results.

**Table 2:** Associated diet factors for dental erosion.

Summary of Factors	Meta-analysis Studies Included	Total Number of Case	OR(95% CI)
Soft drink	1,2,3,4,5,6,7,8,9	8877	2.41(2.03–2.85)
Juice	1,4,5,6,7,8	7041	0.90(0.25–3.24)
Sports drink	1,2,4,5,7	5652	1.58(0.88–2.85)
Milk	1,2,7,8	3387	0.67(0.11–4.01)
Vitamin C	2,3,4,8	3975	1.16(1.10–1.22)
Yoghourt products	2,4,5,7,8	5618	1.05(0.28–3.96)

**Figure 2:**
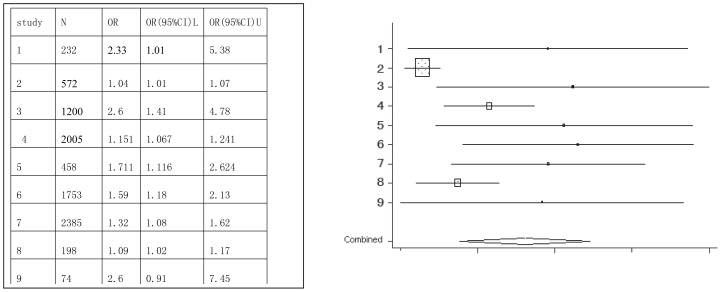
Meta-analysis for the association between soft drink risk and dental erosion.

**Figure 3:**
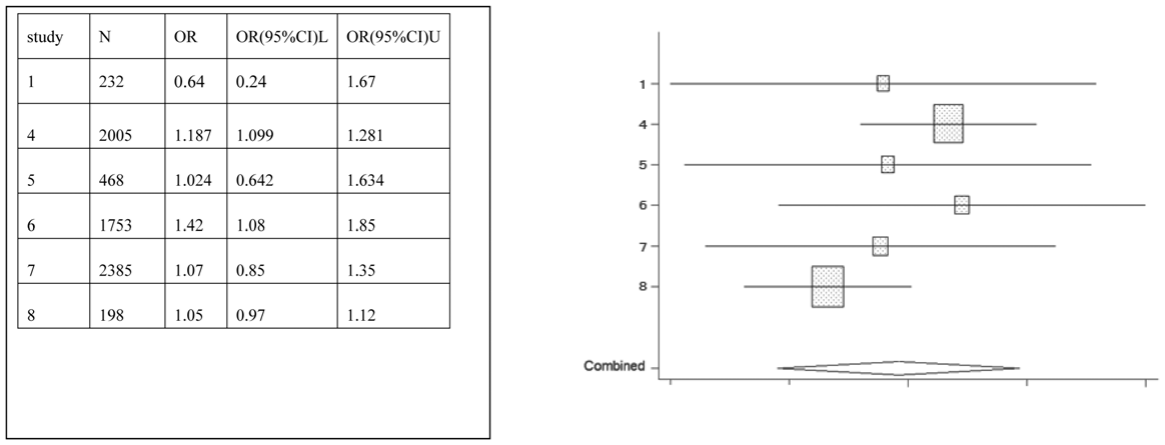
Meta-analysis for the association between juice risk and dental erosion.

**Figure 4:**
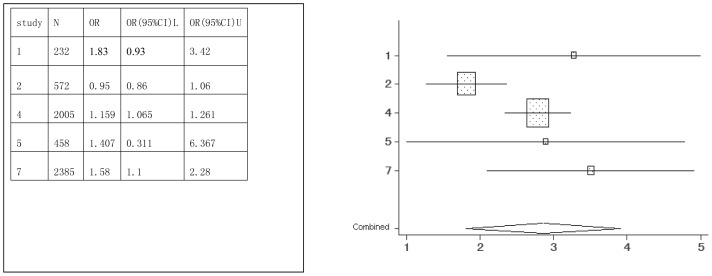
Meta-analysis for the association between sports drink risk and dental erosion.

**Figure 5:**
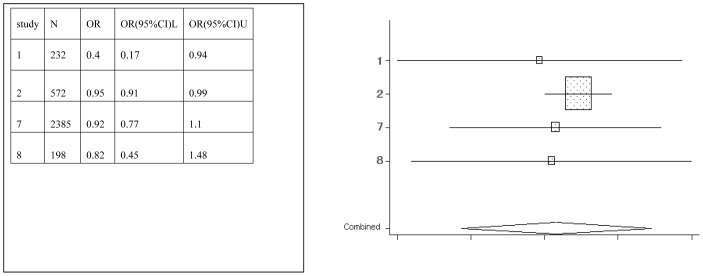
Meta-analysis for the association between milk risk and dental erosion.

**Figure 6:**
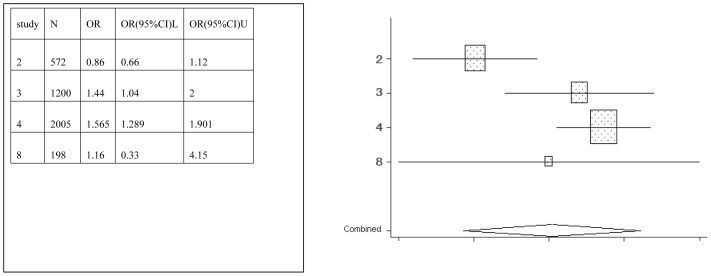
Meta-analysis for the association between vitamin C risk and dental erosion.

**Figure 7:**
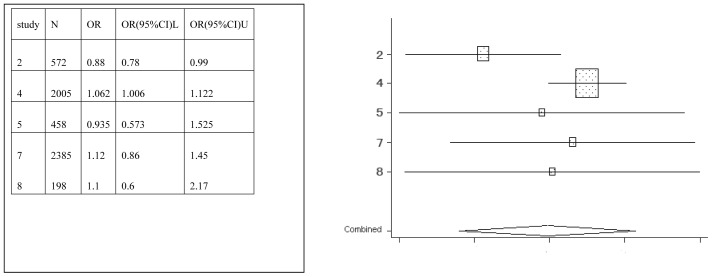
Meta-analysis for the association between yoghourt products risk and dental erosion.

Forest plots were created, which show the effect estimate (Figures 2, 3, 4, 5, 6, 7).

## Results

Figure 1 presents the flow of papers through the study selection process. The literature search produced 417 citations. Review of titles and abstracts resulted in the selection of 12 papers, among which 9 met the inclusion criteria. Selected characteristics of the 9 included articles were reported individually in Table 1. Most studies were conducted in UK and Asia.

When interpreting the result from this meta-analysis, one must consider the issue of heterogeneity among studies. In the process of meta-analysis of 6 associated factors, the *P* value of the heterogeneity test in the overall analysis was >0.10, since a Mantel and Haenszel method was used. In most instances, however, results were fairly consistent in the direction of the effect (ie, pointing toward an increase or decrease in risk), even though studies differed in the estimation of the effect size.

Table 2 provides the ORs and 95% CIs for each associated factor. Soft drink (OR = 2.41, 95%CI = 2.03–2.85) and vitamin C (OR = 1.16, 95%CI = 1.10–1.22) were the risk factors for dental erosion. And juice (OR = 0.90, 95%CI = 0.25–3.24), sports drink (OR = 1.58, 95%CI = 0.88–2.85), milk (OR = 0.67, 95%CI = 0.11–4.01) and yoghourt products (OR = 1.05, 95%CI = 0.28–3.96) were not associated with dental erosion.

## Discussion

Although many studies have investigated associated factors for dental erosion, a comprehensive and quantitative summary has been lacking and several studies presented crude ORs only despite the possible confounders on the association between each factor and the risk of dental erosion.

This review underscores factors that associated to dental erosion, and although most associations are not large, these factors are common for the association with dental erosion. Soft drink was associated with about 2.4 fold risk of dental erosion in this meta-analysis. Soft drinks, excluding milk and water could cause damage to the teeth for two reasons: Firstly, the low pH and high titratable acidity. Secondly the sugars in drinks are metabolized by plaque microorganisms to generate organic acids that bring about demineralization. Erosion is due to the loss of the outermost surface of enamel and occurs when the surface pH falls below 5.5 [27]. For instance, the mean pH of Coca Cola samples analyzed was 2.30, while the mean calcium and fluoride ion concentration were 0.58 and 0.066 respectively. The low pH as well as the low calcium and fluoride ion concentration indicate the high erosive potential [28]. So intervention measures should be taken to prevent or reduce dental erosion from diet factors. Dental professionals should educate the patient about the consequences of frequent soft drink consumption and provide positive suggestions to minimize the risk. Public health providers should guide the people especially teenagers and children to limit the intake of soft drinks. Oral health educators should reinforce important practices to soft drink users such as decreasing the time that the soft drink remains in the mouth.

Chewing vitamin C tablets were significantly associated with the development of tooth wear. Vitamin C (ascorbic acid) has low pH and high titratable acidity. In the light of World Health Organization’s recommendations to consume at least 400 g fruit per day to prevent the onset of chronic conditions, people should be guided by public health providers and oral health educators to develop reasonable oral health behavior.

Juice, sports drink, milk and yoghourt products were not found to be associated with dental erosion in this present meta-analysis. Milk and Yogurt provides an important source of dietary calcium, phosphate and casein, all of which are known to protect enamel [29]. Since the significant risk factors included soft drinks, the frequent consumption of milk could be considered as a substitute way in diet behavior to prevent dental erosion.

The present meta-analysis had several limitations that must be taken into account. First, the number of available studies that could be included in this meta-analysis was moderate. Therefore, the results could be influenced by the factors like random error. Second, though we have searched as many publications as we could by means of various searching approaches, these results may be biased by the relatively small number of subjects.

This meta-analysis provides the first comprehensive evidence-based assessment of relevant factors on dental erosion and intends to drive more attention to the necessity of further community education concerning the loss of the hard tooth substances due to erosion. Erosions are mainly caused by excessive consumption of erosive food and drinks especially among children. In addition, considering the limitation of this study, further larger studies should be considered and investigated to validate the current meta-analysis.
