# Correction: Dietary Factors Associated with Dental Erosion: A Meta-Analysis

**DOI:** 10.1371/journal.pone.0161518

**Published:** 2016-08-19

**Authors:** Haifeng Li, Yan Zou, Gangqiang Ding

In Table 1, the location for Waterhouse PJ et al. (2008) is incorrect. Please see the corrected Table 1 here.

**Table 1:** Summary of studies for dietary factors associated with dental erosion.

Study	Author, Reference	Year	Location	Sample Size Number	Female(%)	Mean or Median Age of Study Population
1	Nahás Pires Corrêa MS et al[16]	2011	Brazil	232	50	9.5
2	Aidi HE et al[17]	2011	Netherlands	572	49	11.9
3	Ratnayake N et al[18]	2009	Sri Lanka	1200	53	17
4	Chen YG et al [19]	2009	China	2005	48	5,12
5	Waterhouse PJ et al[20]	2008	Brazil	458	NR	13.8
6	Dugmore CR et al[21]	2004	UK	1753	49	12
7	Milosevic A et al[22]	2004	UK	2385	52	14
8	Tarsitsa Gatou et al[23]	2011	GREECE	198	50	6.37
9	Milosevic A, et al[24]	1997	UK	74	NR	15

NR indicates not reported
